# The Women’s Health Initiative cancer survivorship clinic incorporating electronic patient-reported outcomes: a study protocol for the Linking You to Support and Advice (LYSA) randomized controlled trial

**DOI:** 10.1186/s40814-022-01186-x

**Published:** 2022-11-10

**Authors:** Noreen Kearns, Laia Raigal-Aran, Kate O’Connell, Andrea Davis, Katie Bermingham, Seamus O’Reilly, Dearbhaile C. Collins, Mark Corrigan, John Coulter, Vicki Cleary, Samantha Cushen, Aileen Flavin, Fiona Byrne, Aisling O’Grady, Deirdre O’Neill, Aileen Murphy, Darren Dahly, Brendan Palmer, Roisin M. Connolly, Josephine Hegarty

**Affiliations:** 1 Catherine McAuley School of Nursing and Midwifery, University College Cork, Cork, Ireland; 2 Cancer Research @UCC, College of Medicine and Health, University College Cork, Cork, Ireland; 3 Department of Medical Oncology, Cork University Hospital, Cork, Ireland; 4 Department of Nutrition and Dietetics, Cork University Hospital, Cork, Ireland; 5 Department of Medical Oncology, South Infirmary Victoria University Hospital, Cork, Ireland; 6 Department of Academic Surgery, Cork University Hospital, Cork, Ireland; 7 Department of Gynaecology Oncology, Cork University Maternity Hospital, Cork, Ireland; 8 School of Food and Nutritional Sciences, University College Cork, Cork, Ireland; 9 Department of Radiation Oncology, Cork University Hospital, Cork, Ireland; 10 Department of Economics, Cork University Business School, University College Cork, Cork, Ireland; 11 HRB Clinical Research Facility, University College Cork, Cork, Ireland; 12 School of Public Health, University College Cork, Cork, Ireland

**Keywords:** Cancer survivorship, Symptom management pathways, Supportive care, Nurse-led, Dietitian resource, Electronic patient-reported outcomes (ePROs)

## Abstract

**Background:**

The improved survival rate for many cancers in high-income countries demands a coordinated multidisciplinary approach to survivorship care and service provision to ensure optimal patient outcomes and quality of life. This study assesses the feasibility of introducing a Women’s Health Initiative cancer survivorship clinic in Ireland.

**Methods:**

The trial https://spcare.bmj.com/content/9/2/209.short comprises an intervention and control arm. Two hundred participants will be recruited. Key eligibility (1) women with early-stage hormone receptor-positive breast or gynecologic cancer (cervix or endometrial), within 12 months of completion of primary curative therapy, and (2) access to the Internet.

The complex intervention comprises a nurse-led clinic targeting symptom management through a trigger alert system, utilizing electronic patient-reported outcome (ePRO) assessments at baseline, and 2, 4, 6, 8, 10, and 12 months. It also includes input from a dietitian monitoring diet and nutritional status.

The control group will receive their usual care pathway standard of care and attend the cancer survivorship clinic and complete ePRO assessments at the start and end of the study.

The primary endpoint (feasibility) includes the proportion of enrolled participants who complete baseline and follow-up ePRO surveys and partake in health professional consultations after ePRO data triggers. Secondary endpoints include changes in cancer-related symptom scores assessed by ePROs, health-related Quality of Life Questionnaire (QLQ) scores, Appraisal Self-Care Agency-R scores, and adjuvant endocrine therapy medication adherence. A process evaluation will capture the experiences of participation in the study, and the healthcare costs will be examined as part of the economic analysis.

Ethical approval was granted in December 2020, with accrual commencing in March 2021.

**Discussion:**

This protocol describes the implementation of a parallel arm randomized controlled trial (RCT) which examines the feasibility of delivering a Cancer Survivorship Clinic. The ePRO is an innovative symptom monitoring system which detects the treatment-related effects and provides individualized support for cancer survivors. The findings will provide direction for the implementation of future survivorship care.

**Trial registration:**

ClinicalTrials.gov, NCT05035173. Retrospectively registered on September 5, 2021

**Supplementary Information:**

The online version contains supplementary material available at 10.1186/s40814-022-01186-x.

## Background

While cancer is one of the leading causes of death globally, and the number of people diagnosed with cancer continues to rise annually, the survival rate for many cancers is improving. Advances in early detection, effective therapies, and supportive care in many parts of the world have led to improvements in 5-year survival rates, with growing numbers living with and beyond cancer [1]. The 5-year global prevalence of all cancers in Europe was approximately 13.5 million people in 2020, representing almost 27% of all cases globally [2]. Given such prevalence data, there is a need for highly coordinated care to ensure both optimal patient outcomes and quality of life [3]. However, to date, an internationally coordinated, systematic approach for survivorship care and service provision underpinned by clinical evidence is lacking [4], and there is no consensus on a generic practical approach to organizing survivorship care in Europe [5].

The many and varied needs of patients on the cancer survivorship trajectory require a thorough understanding of their cancer-related problems [6] and timely access to survivorship services using a holistic need assessment approach; incorporating physical, psychological, and functional needs to inform care [4]. The value of and need for improved collection of patient-reported outcomes (PROs) and data on health-related quality of life (HRQOL) during cancer survivorship has been highlighted by Lagergren et al. [5]. PROs are reports of the status of a patient’s health condition that comes directly from the patient, i.e., self-reported, and are used to assess concepts that may be narrow (e.g., pain intensity) or broad (e.g., HRQOL) [7]. There is evidence of a growing trend towards the development of electronic methods of data collection enabling remote real-time monitoring of patients [8]. Advantages of using computer-based electronic patient-reported outcomes (ePROs) include improved communication between patients and service providers, timelier outcome measurement, improvements in survival, earlier responsiveness to symptoms, better symptom management, and higher levels of patient satisfaction [9–14].

This paper describes an ongoing single-center randomized controlled trial (RCT), assessing the feasibility of introducing a Women’s Health Initiative (WHI) cancer survivorship clinic in a university hospital setting in the Republic of Ireland. The clinic is nurse-led and multidisciplinary and incorporates ePRO technology for women with early-stage hormone receptor (HR)-positive breast or gynecologic cancer. The study will evaluate the feasibility of the clinic by testing two hypotheses. Firstly, the introduction of a WHI cancer survivorship clinic (complex intervention) into routine follow-up care will be feasible. And secondly, female cancer survivors who participate in a survivorship clinic intervention incorporating ePRO collection and targeted symptom management pathways (intervention arm) are more likely to experience improvements in symptom burden and HRQOL compared to those who did not partake in the targeted symptom management pathways (control arm).

## Methods/design

### Study design

The study design is a conventional two-group, parallel RCT with an intervention and control arm [15, 16]. This unblinded RCT assesses the feasibility of introducing a nurse-led cancer survivorship clinic incorporating symptom management through ePROs and a dietician-led nutrition component in patients with early-stage HR-positive breast and gynecologic cancer post-primary therapy.

Nurse-led models of care entailing nurses with the appropriate skills and training in survivorship care have been shown to be particularly important with respect to meeting patient’s needs for follow-up care and support, including symptom management [4, 17]. Moreover, there is clear evidence that diet, nutrition, and physical activity can reduce the risk of certain cancers and more evidence is emerging about the benefits of such for cancer survivors [18, 19]. Therefore, this study incorporates a dietitian-led component to advise on a healthier diet and lifestyle to reduce the risk of cancer recurrence.

### Research ethics

This study will be conducted in accordance with the Declaration of Helsinki, the applicable sections of ICH E6 Good Clinical Practices [20] and the terms of approval of the responsible Clinical Research Ethics Committee of the University’s Teaching Hospital. Full ethical approval for the trial was granted by the Clinical Research Ethics Committee in December 2020 (ECM 4 (y), October 20, 2020). All subsequent amendments to the protocol which impacted or may impact on the conduct of the study have been or will be submitted as amendments for approval to the ethics committee. The most recent protocol amendment was approved in March 2022 (ECM 3 (1), May 04, 2022. The study is registered with ClinicalTrials.gov with a trial registration number of NCT05035173. The manuscript is reported using the Standard Protocol Items: Recommendations for Interventional Trials (SPIRIT) 2013 Checklist for a clinical trial protocol [21] (see Additional file 2).

### Study participants

Women with early-stage HR-positive breast cancer or gynecologic cancer within 12 months of completion of primary curative therapy are eligible. Detailed inclusion and exclusion criteria are outlined in Table 1.

**Table 1:** Eligibility criteria

Inclusion criteria	Exclusion criteria
• Aged 18 years or older	• Not treated with curative intent
• Diagnosed with early-stage breast or gynaecologic cancer within 12 months of completion of primary curative therapy - Breast cancer: Stage I-III hormone receptor-positive (defined as estrogen receptor and/or progesterone receptor ≥ 1%) and HER2-negative per ASCO-CAP guidelines on or recommended to commence adjuvant endocrine therapy during the study period - Cervical cancer: Stage I to III treated with curative intent • Endometrial cancer: treated with curative intent adjuvant radiotherapy +/- chemotherapy	• Diagnosed with premalignant disease (e.g., Ductal Carcinoma In-Situ DCIS/Lobular Carcinoma In-Situ LCIS)
• Have access to the internet	• Persons who, in the opinion of the Principal Investigator, researcher or supervising clinician, are unable to engage adequately with the study protocol (e.g., inability to travel to the hospital site for in-person visits, unable to communicate fluently in English)
• Ability to provide written informed consent	• Recent participation (within 12 months) in a similar study or programme involving a lifestyle intervention (e.g., diet, exercise, survivorship), based on the discretion of the Principal Investigator
• Ability to read and understand English	

### Recruitment and screening

Participants will be recruited across a university hospital group comprising of three participating hospitals within a health service region in the Republic of Ireland. A target sample of 200 participants meeting the predefined inclusion criteria will be recruited. Potential participants will be screened by the oncology clinical care team or research team to determine eligibility and all those who meet the inclusion criteria are invited to participate. The study nurse will log all potentially eligible women and provide them with oral information about all aspects of the study, including the 2 monthly electronic surveys. A follow-up email containing written information about the study in the form of the ethics-approved participant information leaflet and consent form will be sent. If potential participants express interest in the study informed consent is sought. All study participants who provide written informed consent either in person, at a clinic visit, or by email or post, are enrolled in the study. The Castor Electronic Data Capture (EDC) platform (https://www.castoredc.com/) will be used to collect and store the study data. Study participants will be facilitated with access to the ePRO measurement system via Castor EDC.

Recruitment will take place over an 18-month period (March 2021 to September 2022). The study duration for each participant is 12 months. Strategies being taken to reach the target sample size of 200 include readily available study brochures and contact details, the use of social media and print media, and education of oncology staff in the participating hospitals about the study.

### Randomization

Following consent and enrolment, participants will be randomized and assigned a unique record ID and access to the ePRO measurement system. Randomization of participants will be stratified by a cancer diagnosis, with one randomization list for patients with breast cancer and one for patients with cervical and endometrial cancer. Participants will be randomized in a 1:1 ratio to either the intervention group or to the control group, using a computer-generated randomization list using randomly sized blocks of size 4, 6, or 8. Allocation will be done by the study’s principal statistician, via Castor EDC, a secure, computerized database, and the study investigators do not have access to the allocation sequence. It is not possible for research team members to be blinded to participants’ group allocation as they will be performing the intervention sessions relevant to each group. However, allocations are only revealed to the research team once a patient has unambiguously consented and enrolled on the trial, and allocations cannot be altered in the database once a patient is enrolled. Furthermore, it is not possible to blind participants to group allocation as they will know which group they are in.

### Intervention group

There are two distinct disciplinary components of the complex survivorship intervention, one is the nurse-led clinic targeting improved symptom management, and the other is a dietitian-led component focused on enhancing diet and nutrition (Table 2). The intervention group will have access to the services offered by the WHI cancer survivorship clinic at the start and end of the study period and when needed during the 12-month study period. Participants in the intervention group will receive a link to a survey via email and undertake ePROs at baseline and at 12 months (end of study). The survey includes a series of assessments concerning symptoms, quality of life, nutrition, and body composition.

**Table 2:** Study calendar

Intervention components	Intervention Group	Control Group
Information about the study (oral and written formats)	x	x
Informed consent	x	x
Enrolment in the study	x	x
Clinical and socio-demographic data collection	x	x
ePROs collected at baseline and end-of-study	x	x
A baseline clinic visit involving:		
- one-on-one consultation with the study nurse	x	x
- one-to-one consultation with the study dietitian and conduct of dietary and body composition assessments	x	x
- a study resource folder containing “Survivorship Personal Treatment Care Plan” and recommended support resources about survivorship	x	x
- an “Education and Symptom Management Pathway”	x	
- “Personalized Nutrition Care Plans” containing diet education and nutritional counselling for participants at nutritional risk	x	
Ongoing monitoring of cancer related symptoms collected via ePRO symptom questionnaires at 2, 4, 6, 8, and 10 months	x	
Access to a trigger-initiated symptom management pathway(s) or a six-monthly follow-up phone call with the study’s nurse (if no triggers)	x	
Ongoing monitoring of weight, physical activity and changes in nutritional risk collected via ePRO symptom questionnaires at 2, 4, 6, 8, and 10 months	x	
Access to a trigger-initiated nutritional management pathway	x	
End of study clinic visit involving:		
- one-on-one consultation with the study nurse	x	x
- one-to-one consultation with the study dietitian and repeat dietary and body composition assessments	x	x
Feasibility process evaluation surveys, and interviews or focus groups (post-intervention)	x	x
Measurement of resource utilization	x	x

*ePROs* Electronic patient reported outcomes

Once the ePRO baseline assessment survey is completed, participants in the intervention arm will be invited to attend an initial clinic visit entailing consultations with the study nurse and study dietitian. Participants will receive a study resource folder containing routine standard information and resources about survivorship. They will also receive a “Survivorship Personal Treatment Care Plan” depending on their type of cancer and an “Education and Symptom Management Pathway” which is tailored according to the results of the baseline ePROs. There will be ongoing monitoring of intervention group participants’ symptoms during the study period (months 2, 4, 6, 8, 10), and a trigger system will notify the nurse and/or dietitian of worsening or severe ePRO scores. This trigger system or clinical judgment will prompt symptom evaluation and management in-between routine clinic visits by the study nurse for the intervention arm. The type of symptom support available to intervention group participants will range from supported self-management (i.e., targeted information and advice, telephone support) to onsite clinic-based specialist advice and support, or referrals as specified within a symptom management pathway. Symptom pathways in this study will be available for common symptoms assessed in the ePRO measures such as joint pain, depression, anxiety, vaginal dryness, dyspareunia, hot flashes, cognitive impairment, and fatigue. In the event of no trigger alerts, the study nurse will follow up with intervention participants via a phone call at 6 months, to clarify as per the completed 2 monthly surveys that all is going well. Routine standard of care visits will continue, with participants’ referring team of health care professionals, throughout the study as per international guidelines.

The dietitian consultation at baseline provides intervention participants with “Personalized Nutrition Care Plans” involving diet education and nutrition counseling, based on their dietary intake and diet quality assessment ePRO scores. History of alcohol intake and physical activity levels will also be discussed at the initial visit. There will be ongoing self-reported monitoring of intervention participants’ weight and nutritional status during the 12-month intervention period. Participants in the intervention group will be given personal nutrition care plans and nutritional counseling if they trigger on the ePRO system or based on clinical judgment.

### Control group

The control group is an active comparator receiving the current standard of care. Control group participants, however, will also attend the WHI cancer survivorship clinic to have consultations with the nurse and dietitian at the start (baseline) and end of the study periods. These clinic visits are not currently offered as standard of care, and we hope the clinic visits will encourage ongoing participation after randomization At the initial clinic visit, they will receive a “Survivorship Personal Treatment Care Plan” depending on their type of cancer and survivorship resource information. Baseline and end-of-study assessments of dietary intake and body composition measurements will be recorded during these two clinic visits. The control arm participants will not complete ePROs or attend the clinic in-between baseline and end-of-study time points. During this period, their care will involve routine surveillance via their usual care pathway as per international guidelines comprising of multidisciplinary oncology teams and/or other specialists or services as required.

### Participant flow through the study

**Fig. 1:**
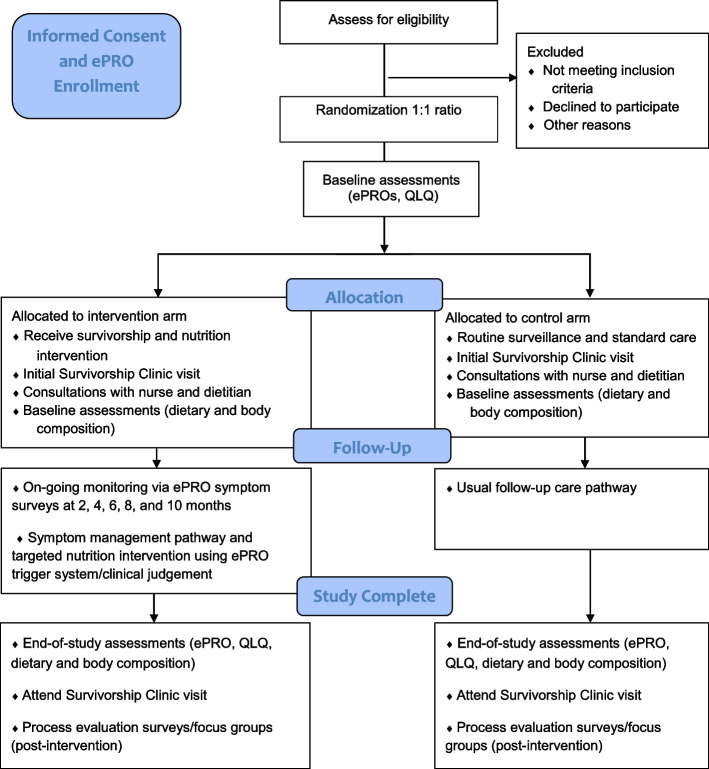
Women’s Health Initiative cancer survivorship clinic RCT Trial CONSORT flow diagram

Figure 1 depicts an overview of study participants using the Consolidated Standards of Reporting Trials CONSORT flow diagram [22].

To promote participant retention and completion of the 2 monthly surveys, an automatic reminder will be emailed if a completed ePRO survey is not returned within 24 h. There will be a second reminder via a telephone call from the study nurse within 72 h to encourage completion. After 7 days, the ePRO symptom survey will be locked. If a study participant does not complete two consecutive ePRO surveys, or if the participant decides to cease involvement, the study nurse will make contact to check in with them regarding their reasons for not continuing in the study and inform them that they will be withdrawn. We will use relevant information from patient records and discussions with the referring health care professional team and/or the participant to determine the reason(s) for discontinuation. The reasons for the discontinuation of participation (Table 3) in the trial will be recorded in the case report form.

**Table 3:** Reasons for Discontinuation

At the participant’s own request	
Death	
Intercurrent illness or condition that would, in the judgment of the Principal Investigator, significantly affect assessment of clinical status	
Evidence of disease recurrence during the study	
The study is terminated for any reason	
The participant withdraws consent for follow-up	
Non-completion of two consecutive ePRO surveys	

*ePRO* Electronic patient reported outcome

### Study objectives

The primary objective of the study, namely an evaluation of the feasibility of the Women’s Health Initiative cancer survivorship clinic, will be assessed according to several feasibility outcomes, including the proportion of enrolled participants who complete the baseline and follow-up ePRO surveys, and the proportion of participants who partake in healthcare professional consultations following ePRO data triggers; the proportion of participants that require medical review and the timeframe involved; the average consultation time; the number of participants enrolled in the clinic; and extra health care professional time and resources needed to run the intervention. Secondary endpoints include changes in cancer-related symptom scores assessed by ePROs, health-related Quality of Life Questionnaire (QLQ) scores, Appraisal Self-Care Agency-R scores, and adjuvant endocrine therapy medication adherence.

### Measures

Table 4 (see Additional file 1) provides an overview of the study outcomes and data collection time points. The feasibility trial will evaluate the feasibility of introducing a cancer survivorship clinic. The study will also survey the effect of the intervention on key ePROs, i.e., HRQOL and symptoms experienced. The five targeted issues that the trial is focusing on are fatigue, hot flashes, fear of cancer recurrence, vaginal discomfort, and diet and weight gain/loss concerns. The European Organization for Research and Treatment of Cancer Quality of Life (EORTC QOL) patient-reported core and disease-specific measures will be used pre- and post-intervention (i.e., at baseline and end of study) to assess HRQOL. Health-related quality of life (QLQ) scores assessed by the EORTC core, and breast cancer, cervical cancer, and endometrial cancer modules will be analyzed by linearly transforming scores to a 0–100 scale, with a high or healthy level of functioning representing a high functional score. Other pre- and post-data being collected includes self-care agency. Appraisal Self-Care Agency R-scores will be reported, with a higher score representing a better self-care agency.

The Symptom Survey ePRO package includes items from both the patient-reported outcomes version of the Common Terminology Criteria for Adverse Events (PRO-CTCAE) [7] and the Patient-Reported Outcomes Measurement Information System (PROMIS) [23, 24]. Participants in both study arms will complete ePRO symptom questionnaires at baseline and end of study timepoints. The ongoing monitoring of symptoms is for the intervention arm only occurring at 2, 4, 6, 8, and 10 months. Higher PRO-CTCAE responses represent worse functioning, indicated by higher frequency, greater severity, and/or more interference. Higher PROMIS T-scores indicate more symptoms, indicated by more of the domain being measured.

In addition to the PRO-CTCAE and PROMIS items, the following data will also be collected on an ongoing basis: fear of cancer recurrence, self-reported adjuvant endocrine therapy medication adherence, weight, and direct health care resource use. Adjuvant endocrine therapy medication adherence will be assessed by analyzing participants’ self-reporting data.

Both arms of the study will partake in nutritional and physical assessments at baseline and end-of-study timepoints conducted by a dietitian. Dietary intake assessments include two 24-H Dietary Recalls (24HDR) [25–27] and a Food Frequency Questionnaire (FFQ) [28]. By using a combined dietary method for assessment, the calculation of an individual’s usual dietary intake will be subject to less measurement error [29, 30]. The first 24HDR assessment will be done face to face in the clinic by the study dietitian to capture a weekday dietary intake. The second 24HDR will also be done by the study dietitian, using T-Pro, a telehealth platform, to capture a weekend day dietary intake alongside the self-reported FFQ (which will capture the previous 12 months of dietary intake). Portion sizes will be validated through the use of a food atlas. Dietary quality will be measured using the World Cancer Research Fund/American Institute for Cancer Research (WCRF/AICR) [31] standardized scoring system. Physical assessments of body weight, height, body mass index (BMI), body composition, waist circumference, and muscle strength will be recorded. Participants’ nutritional status will be monitored over the 12-month period via the ePRO symptom surveys where data relating to changes in body weight, concerns about weight gain, nutritional risk, and physical activity will be collected at baseline and 12-month timelines for the control group, and at baseline, months 2, 4, 6, 8, 10, and 12 for the intervention group. The Malnutrition Screening Tool (MST) [32] will be used to identify participants at risk of malnutrition. The Nordic Physical Activity Questionnaire NPAQ-short [33] will be used to measure participants’ physical activity levels.

For the process evaluation, information will be sought on the user experience of the clinic and the computer-based ePROs. This will be done through the administration of a short post-intervention survey to participants from both groups, containing questions on usability and satisfaction. Other quantitative descriptive data will be compiled from the study’s records in order to describe what was delivered in terms of the intervention, and to whom, focusing in particular on the questions of fidelity, dose, and reach. In addition, qualitative data will be gathered at the end of the study from a subset of participants from both study arms who will be invited to take part in semi-structured interviews or focus groups to give their opinions of involvement in the WHI cancer survivorship clinic. Interviews or focus groups with implementers of the intervention, namely health care professionals, and key stakeholders, will also be conducted to discuss perceived facilitators and barriers to the roll-out of the clinic.

The economic evaluation will consider the implementation costs of the intervention, estimated using trial information and health care resource utilization collected via a researcher-designed tool. Descriptive data regarding the number of resources used by each participant will also be presented, based on analyzing data from patient hospital record forms, case-report forms, use of resources forms, and patient diaries. Effectiveness will be measured in terms of quality-adjusted life-years (QALYs). EQ-5D-5L-QALY scores will be reported, with a high score representing high functionality problems.

### Adverse events

This study has been deemed low risk by the study sponsor based on the stated intervention (nurse-led clinic intervention with serial ePRO data collection), and therefore, study-related adverse events are not envisaged. There are no anticipated excess risks to participants receiving the intervention, and all participants are guaranteed access to the current standard of care. However, if study participation results in any event that has negative consequences for the participant, the study nurse will discuss and record this with the Principal Investigator. This information will be recorded by the Principal Investigator in the Castor EDC platform and reported to the sponsor of the study, i.e., the university, and the University’s Teaching Hospital Research Ethics Committee, as per the study protocol.

### Data management and analysis

The study’s protocol is accompanied by a Data Management Plan. For confidentiality purposes, a study-specific ID code will serve as a unique identifier on the case-report forms for all study participants. The codes will be stored separately from the main study database and other study documentation. This unique ID subject code will be linked to the participant’s identifying information by a participant identity list maintained by the study’s Principal Investigator in a secure location at the clinical site of the study. The list of ID codes will not be forwarded to any third party. The purpose of the ID code is to facilitate subsequent follow-up of participants in the event of requiring symptom management intervention.

An on-site Trial Master Folder (TMF) containing confidential data in the form of hard copies of regulatory documents and participant-related information will be kept in a locked cabinet in a locked room, accessible only by the study’s research team members. An electronic TMF containing confidential regulatory documents, participant-related information, and patient public interactions will be maintained in a dedicated database in a secure location. This confidential electronic data will be stored and backed-up monthly in encrypted folders on a health service laptop, and in the validated web-based nutritional analysis software tool, Nutritics (https://www.nutrics.com/app/#). Access to the database will be controlled at the level of the individual with specific roles assigned, with concomitant access rights (e.g., data manager, data entry, auditor). At the end of the study, all anonymized data will be available to the study’s principal statistician, while researchers nominated by the Principal Investigator will also be able to access the final trial dataset.

The study’s primary data catalog, encompassing primary participant data (case-report forms) and participant responses to electronic surveys, will be collected and managed using the Castor EDC platform. This platform meets national and international guidelines with respect to data privacy and data protection standards. Once data entry is finalized, study data will be assessed for incompatible, discrepant, or clinically implausible values. Outlying values for all distributions, in isolation and over time, will be identified. Any concerning data will be reconciled against the original source data. Following the completion of cleaning, the database will be locked.

### Sample size

Given that the main objective of this study is to evaluate the feasibility of introducing a survivorship clinic, a pragmatic approach was taken in determining the sample size for this trial. The sample size target was therefore based on the recruitment of the largest possible sample given the numbers of potentially eligible patients attending the institution during the time frame of the study and funding availability. We determined that this would be approximately 200 participants (140 participants with early-stage breast cancer and 60 participants with cervical or endometrial cancer). This pragmatic approach maximizes the ability to identify barriers to implementation of the clinic and collect information in parallel arms that will be required to properly design a next step efficacy study [34, 35] (e.g., robust estimation of estimator variability), even for binary outcomes [36]. With respect to the feasibility outcomes (see below), the 95% confidence interval around a proportion equal to 0.50 at this sample size would be 0.43 to 0.57 (i.e., a margin of error of 0.07) and provide 50% power to declare feasibility when the true rates are ≥0.5 and 80% power when they are ≥0.58 (while preserving the false positive rate at 5% based on a single-sided hypothesis test).

### Statistical analyses

The primary objective of the study, namely an evaluation of the feasibility of the Women’s Health Initiative cancer survivorship clinic, will be assessed according to several outcomes, including the proportion of enrolled participants who complete the baseline and follow-up ePRO surveys, and the proportion of participants who partake in healthcare professional consultations following ePRO data triggers; the proportion of participants that require medical review and the timeframe involved; the average consultation time; the number of participants enrolled in the clinic; and extra health care professional time and resources needed to run the intervention. Uncertainty in each of the figures pertaining to the feasibility outcomes will be conveyed with 95% confidence intervals and compared to progression criteria to guide decision making about the feasibility of a definitive trial. More specifically, progression to a fully powered trial will be dependent on achieving at least 50% of participants in the intervention arm completing the baseline and follow-up ePRO surveys, and at least 50% of participants who have ePRO triggers linking with or partaking in a health professional consultation after the ePRO data trigger, if required.

While this is a feasibility study, we will also take advantage of the collection of ePROs (described above) to estimate the efficacy of the intervention using generalized linear models with adjustment for baseline outcome and key prognostic covariates in the intention to treat the sample. Prognostic covariates are pre-specified in the Statistical Analysis Plan which accompanies the study protocol, and any adjustments will be made a priori. Such information will contribute to the design of the definitive trial (if feasibility is established). Any estimates reported alongside indicators of uncertainty (*p* values and confidence intervals) will be presented in a purely exploratory manner, with no decisions about the efficacy of the intervention inferred in any reports of the research.

Data analyses will be conducted and/or supervised by the study’s principal statistician, under established quality systems and standard operating procedures, and in accordance with ICH E9 Statistical Principles for Clinical Trials [37] and ICH E6 Good Clinical Practices [20], and following a detailed statistical analysis plan that will be pre-registered prior to database lock. Analyses will be conducted using the R Project for Statistical Computing and the RStudio integrated development environment (IDE) statistical software packages. The trial reporting will be done following Consolidated Standards of Reporting Trials CONSORT and the CONSORT addendum for pilot and feasibility trials [38]. Missing data will be evaluated, based on what is observed, and dealt with in whatever manner deemed most appropriate, based on current best practices.

Nutritics will be utilized to capture dietary intake information, and data from the multiple 24HDR assessments will be analyzed using the UK’s composition of foods data from McCance and Widdowson’s integrated dataset [25], the United States Department of Agriculture (USDA) Food Composition Database [26], and the Irish food composition database [27]. Data concerning changes in nutritional status will be complemented by nutrient data from the European Prospective Investigation into Cancer Study EPIC-Norfolk Food Frequency Questionnaire (FFQ) [28] based on version 6 (CAMB/PQ/6/1205). Data gathered via the FFQ will be entered into a validated processing tool, the FFQ EPIC Tool for Analysis (FETA), based on the Compositional Analyses from Frequency Estimates (CAFE) system [28]. Changes in diet quality scores will be based on the WCRF/AICR standardized scoring system [31], with higher scores representing a better diet quality. BMI scores will be calculated based on combined weight and height scores and are part of the WCRF/AICR scoring system. Changes in waist circumference will be calculated based on physical assessments performed by the study dietitian. Segmental body composition scores will be measured by the body composition monitor bioimpedance spectroscopy. Muscle strength scores will be measured in terms of handgrip strength measurements based on the Jamar Dynamometer (Model 091011725), with the handle in the second position as recommended by the American Society of Hand Therapists [39]. Nutritional risk scores will be assessed by the MST [32] with a higher score representing a higher risk of malnutrition.

For the process evaluation, the results of the usability and satisfaction survey will be summarized visually in a table and presented as response distributions (number, percentage). Analysis of the usability and satisfaction survey questions will involve the summation of usability and satisfaction scale scores and examination of these across respondents, with 0.80 as the proposed threshold for defining usability and acceptability, counting only positive endorsements (4=agree, 5=strongly agree) [9, 40]. Qualitative data will be analyzed thematically.

The economic evaluation will consider the potential cost-effectiveness of the intervention by comparing incremental costs and effects of the intervention with the control incremental cost-effectiveness ratio (ICER), in accordance with the national guidelines [41]. Effectiveness will be measured in terms of QALYs. This will be informed by applying the Irish EQ-5D-5L Value Set [42] to the EQ-5D-5L data collected in the trial. A probabilistic sensitivity analysis will be conducted to investigate uncertainty around parameter inputs and results.

### Data monitoring

The university as the study sponsor is the Data Controller responsible for the study. Castor EDC provides for a full audit trail of user actions to be recorded, and data check procedures for Castor EDC will be conducted every 3 months from the start of the recruitment process by the study team. Biannual monitoring reports will be provided to the study’s Sponsor Office, and a Clinical Trial Audit will be provided by the study’s Clinical Research Facility as required from the Sponsor Office. The study team will provide annual reports to the study’s funders as expressed in the funding agreements. Based on the non-medical intervention and low-risk nature of participation in the study, a Data Monitoring Committee was deemed to be unnecessary.

### Dissemination

In consultation with the study funders, it is planned to disseminate the key findings of the feasibility trial after the end of the study whereby anonymized individual participant data sets will be shared in the form of results within peer-reviewed publications, academic conferences, workshops, and seminars. Planned public and patient dissemination outputs include public blogs, media, and outreach engagement events, including press articles and seminars. The protocol is published on the ClinicalTrials.gov website, with trial registration number NCT05035173. Post-study shared outputs including the study protocol, data dictionary, and analysis scripts will be available via the Open Science Framework.

### Patient involvement

The study team members are collaborating with a public and patient involvement (PPI) panel in the design, development, implementation, analysis, and dissemination of the study. The PPI panel includes patient advocates who have had breast or gynecological cancers, and stakeholders including clinical and academic experts, wider members of the multidisciplinary team, community support groups, and the trial’s funders.

## Discussion

With cancer survivor numbers increasing, optimizing individuals’ quality of life is particularly pertinent for health care systems. There is a growing interest in survivorship care as part of the cancer trajectory, based on a patient-centered, multidisciplinary approach across services in acute, primary, community, and voluntary settings. However, follow-up survivorship care continues to be an underdeveloped facet of the cancer trajectory, and many cancer survivors continue to have a range of unmet needs following initial diagnosis and treatment, with symptoms ranging from physiological to psychological, cognitive, social, sexual, and nutritional [5, 43, 44]. The health care system would be able to respond to the varied and unmet needs of cancer survivors in a more coordinated way if a cancer survivorship pathway was formalized, with the aid of mobile solutions in healthcare, for instance in the form of ePROs [45, 46]. This would involve a holistic needs assessment model of care [4], supporting individuals to transition through the various timepoints of their cancer journey, dealing with troublesome and chronic symptoms, and morbidity or comorbidity health problems in a timely manner. It would also encourage the active involvement of participants in their self-care.

In recognition of the specific, ongoing, and unmet needs of cancer survivors, this study will examine the influence of a complex intervention comprising a WHI cancer survivorship clinic on symptom management and HRQOL. As part of the intervention, the use of ePROs for gathering follow-up symptoms and quality-of-life data will be explored as a means of early intervention. Based on the study results regarding the feasibility of implementing such a clinic model, the data generated will be used to support a full-scale appropriately powered RCT of the survivorship clinic, to highlight learning for the design of survivorship clinics, and ultimately to guide the roll-out of similar clinical care interventions for cancer survivors nationally and internationally in the future. Results from this study will assist the routine implementation of follow-up services for women living with and beyond these types of cancer enabling the health care system to address specific or continuing symptoms and optimizing QOL.

A number of limitations need to be considered in terms of generalizability of the findings. First, since the study is a feasibility trial, it is by design, not powered to reliably detect plausible, clinically meaningful differences and relevant effect sizes in symptoms and quality of life outcomes between the intervention and control arms. Second, participants are being recruited from a single-study site, thereby resulting in a limited number of potentially eligible study participants; however, the inclusion of additional sites is being considered. Third, inclusion criteria mean that participants without access to the Internet, and those who are unable to communicate fluently in English are excluded from the study. Fourth, medication adherence is being measured using self-reports, which are not as reliable as electronic monitoring systems or pill counts [47, 48]. Fifth, this RCT has a high risk of confounding factors since it is a complex intervention taking place in a routine clinical setting. Sixth, the burden introduced by the FFQ dietary assessment may reduce the acceptability of the clinic to participants. Finally, a weakness of the FFQ is that it captures the previous a 12-month dietary intake which can be significantly altered due to treatment and side effects and the accuracy of reporting food frequencies and portions requires good participant memory, literacy, and numerical skills.

## Supplementary Information

**Additional file 1: Table S4.** Study outcomes, assessment methods and time points [49–60].

**Additional file 2.** The Standard Protocol Items: Recommendations for Interventional Trials (SPIRIT) Checklist for a clinical trial protocol.

## Data Availability

Not applicable.

## Abbreviations

ASAS-R: Appraisal Self-Care Agency—Revised tool
ASCO-CAP: American Society of Clinical Oncology/College of American Pathologists
BMI: Body mass index
CONSORT: Consolidated Standards of Reporting Trials
CRF: Clinical research facility
DCIS: Ductal carcinoma in situ
EDC: Electronic data capture
EORTC: European Organization for Research and Treatment of Cancer
EORTC QOL: European Organization for Research and Treatment of Cancer Quality of Life
EORTC QLQ-30: European Organization for Research and Treatment of Cancer Quality of Life Questionnaire—core
EORTC QLQ–BR23: European Organization for Research and Treatment of Cancer Quality of Life Questionnaire—breast cancer subscale
EORTC QLQ–CX24: European Organization for Research and Treatment of Cancer Quality of Life Questionnaire—cervical cancer subscale
EORTC QLQ–EN24: European Organization for Research and Treatment of Cancer Quality of Life Questionnaire—endometrial cancer subscale
ePRO: Electronic patient-reported outcome
EQ-5D-5L: The EuroQol (European Quality of Life) Five Dimension Five Level Scale
FFQ: Food Frequency Questionnaire
HER2: Human Epidermal Growth Factor Receptor 2
HRQOL: Health-related quality of life
ICER: Incremental cost-effectiveness ratio
IDE: Integrated development environment
LCIS: Lobular carcinoma in situ
MST: Malnutrition screening tool
NPAQ-short: Nordic Physical Activity Questionnaire
PPI: Public and patient involvement
PRO: Patient-reported outcome
PRO-CTCAE: Patient-reported outcomes version of the common terminology criteria for adverse events
PROMIS: Patient-reported outcome measurement information system
QALYs: Quality-adjusted life-years
QLQ: Quality of Life Questionnaire
RCT: Randomized controlled trial
SPIRIT: Standard Protocol Items: recommendations for Interventional Trials
TMF: Trial master file
WCRF/AICR: World Cancer Research Fund/American Institute for Cancer Research
WHI: Women’s Health Initiative
