# Correction: The Women’s Health Initiative cancer survivorship clinic incorporating electronic patient-reported outcomes: a study protocol for the Linking You to Support and Advice (LYSA) randomized controlled trial

**DOI:** 10.1186/s40814-022-01219-5

**Published:** 2022-12-12

**Authors:** Noreen Kearns, Laia Raigal-Aran, Kate O’Connell, Andrea Davis, Katie Bermingham, Seamus O’Reilly, Dearbhaile C. Collins, Mark Corrigan, John Coulter, Vicki Cleary, Samantha Cushen, Aileen Flavin, Fiona Byrne, Aisling O’Grady, Deirdre O’Neill, Aileen Murphy, Darren Dahly, Brendan Palmer, Roisin M. Connolly, Josephine Hegarty

**Affiliations:** 1 Catherine McAuley School of Nursing and Midwifery, University College Cork, Cork, Ireland; 2 Cancer Research @UCC, College of Medicine and Health, University College Cork, Cork, Ireland; 3 Department of Medical Oncology, Cork University Hospital, Cork, Ireland; 4 Department of Nutrition and Dietetics, Cork University Hospital, Cork, Ireland; 5 Department of Medical Oncology, South Infirmary Victoria University Hospital, Cork, Ireland; 6 Department of Academic Surgery, Cork University Hospital, Cork, Ireland; 7 Department of Gynaecology Oncology, Cork University Maternity Hospital, Cork, Ireland; 8 School of Food and Nutritional Sciences, University College Cork, Cork, Ireland; 9 Department of Radiation Oncology, Cork University Hospital, Cork, Ireland; 10 Department of Economics, Cork University Business School, University College Cork, Cork, Ireland; 11 HRB Clinical Research Facility, University College Cork, Cork, Ireland; 12 School of Public Health, University College Cork, Cork, Ireland

**Correction: Pilot Feasibility Stud 8, 238 (2022)**

**https://doi.org/10.1186/s40814-022-01186-x**

Following publication of the original article [1], the authors reported an error found in the **Methods** section of the Abstract.

The sentence currently reads:

The trial https://spcare.bmj.com/content/9/2/209.short comprises an intervention and control arm.

The sentence should read:

The trial comprises an intervention and control arm.

The original article [1] has been updated.
