# Supplementary figures and images for: The Women’s Health Initiative cancer survivorship clinic incorporating electronic patient-reported outcomes: a study protocol for the Linking You to Support and Advice (LYSA) randomized controlled trial
Source: Pilot Feasibility Stud. 2022 Nov 10;8:238. doi: 10.1186/s40814-022-01186-x (PMC9648029; doi:10.1186/s40814-022-01186-x)

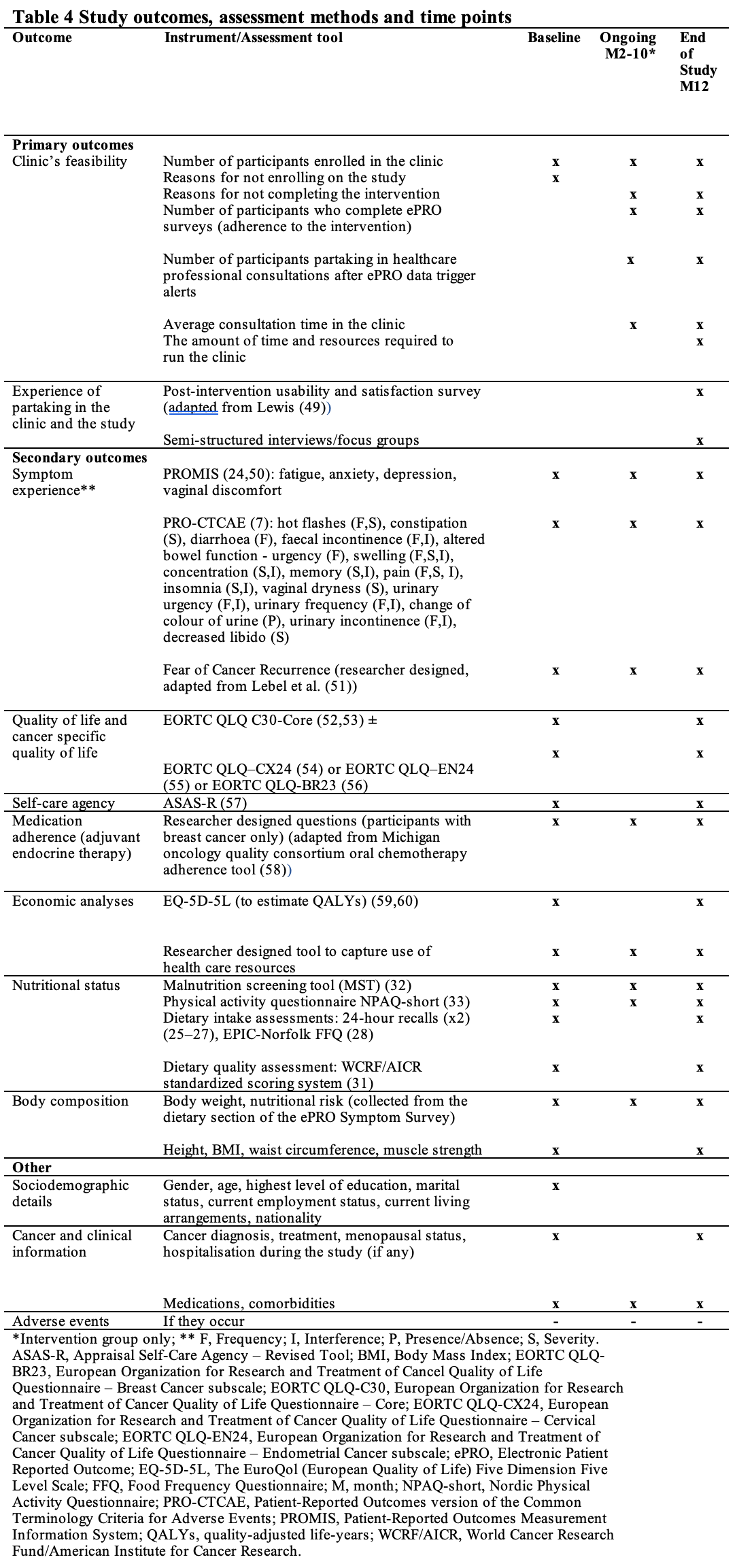

Supplement: Supplementary file 1 — Additional file 1: Table S4. Study outcomes, assessment methods and time points [49–60]. [file 40814_2022_1186_MOESM1_ESM.docx]
